# Mapping the strategies to address the gender gap in youth clinic utilization in Sweden; explorative survey and follow up interviews

**DOI:** 10.1186/s13690-026-01918-y

**Published:** 2026-04-24

**Authors:** Moses Tetui, Emma Bernard Stenmalm, Mazen Baroudi

**Affiliations:** 1 School of Public Health Sciences, University of Waterloo, Waterloo, ON Canada; 2 Department of Epidemiology and Global Health, Umeå University, Umeå, Sweden

**Keywords:** Boys, young men, gender equity, gender-responsive services, intervention, access to care, youth clinics, Sexual and Reproductive Health, Sweden

## Abstract

**Background:**

Despite Sweden’s universalist health model and a long-standing network of youth clinics offering free sexual and reproductive health (SRH) services, boys and young men (BYM) remain significantly underrepresented among clinic users. This gender gap is driven by structural and cultural barriers, including masculine norms, female-oriented service environments, and limited institutional guidance for engaging male adolescents. While some clinics have piloted strategies to improve BYM’s access, these efforts have not been systematically documented or evaluated. The aim of this study was to systematically map and analyze the strategies used by Swedish youth clinics to improve access and participation among BYM.

**Methods:**

This study employed an exploratory mixed-methods design, combining an online survey with follow-up unstructured interviews. The survey was distributed to all 240 youth clinics in Sweden between September and November 2024, with 75 responses representing 110 clinics (45% clinic-level response rate). Quantitative data were analyzed descriptively, while qualitative data from open-ended responses and interviews (24 interviews) were analyzed using inductive content analysis.

**Results:**

BYM accounted for 9.5% of clinic visits, with 87% of clinics expressing dissatisfaction with this level. While 58% of clinics reported using strategies to attract BYM, only 34% of those using strategies evaluated their effectiveness, and institutionalization was limited. Four main strategy categories emerged: (1) staff capacity-building for male-inclusive care, (2) environmental adaptations to create inclusive spaces, (3) digital outreach and marketing to enhance visibility, and (4) collaboration with schools and community settings to reach BYM. Clinics using strategies were more likely to report increased male attendance, though implementation remained inconsistent and often project-based.

**Conclusions:**

This study provides the first national-level mapping of strategies to engage BYM in Sweden and contributes to global discussions on inclusive, adolescent-centered SRH care.

Swedish youth clinics largely operate within a gender-neutral framework that fails to address BYM’s specific needs. A shift toward gender-responsive care, anchored in policy, training, and service design, is urgently needed. National coordination, standardized guidelines, and equity monitoring are essential to ensure that BYM benefit equally from youth health services.

**Trial registration:**

Not applicable.

**Supplementary Information:**

The online version contains supplementary material available at 10.1186/s13690-026-01918-y.

**Table:** 

Text box 1. Contributions to the literature
• This study provides the first national mapping of strategies used by youth clinics in Sweden to improve boys and young men’s use of their services.
• Highlights how gender-neutral service models can unintentionally reinforce inequities in adolescent health access.
• Identifies promising practices, such as staff training, inclusive environments, digital outreach, and school collaboration, that can inform policy and service design.
• Underscores the urgent need for gender-responsive approaches and national coordination to ensure equitable access to sexual and reproductive health services.

## Background

Youth-friendly sexual and reproductive health (SRH) services aim to ensure that all young people—regardless of gender, socioeconomic status, or background—have equitable access to confidential, non-judgmental, and responsive care [1, 2]. While such services have improved health literacy, contraceptive uptake, and STI prevention globally [1, 2], boys and young men (BYM) remain significantly underrepresented both in Sweden and internationally [3]. This underuse is linked to gender norms and service models historically oriented toward women shaping both young men’s reluctance to seek services and providers’ assumptions about whose SRH needs are prioritized [4, 5].

This inequity is driven by structural and cultural barriers, including entrenched gender norms, stigma around male help-seeking, low awareness of SRH services, and the perception that these services are designed “for girls and women [6].” Such barriers are not merely individual; they are reinforced by service models, staffing patterns, and communication strategies that inadvertently favor female clients [7]. As a result, male adolescents’ SRH needs are under-recognized and under-served, with implications for their health outcomes and for broader gender equity goals.

Few evaluated strategies exist to enhance BYM’s participation in youth clinics. A Nordic scoping review [6] identified only one study from Norway examining BYM’s access to youth clinics, where the clinic environment was perceived as female-oriented and unwelcoming [8]. This perception is echoed across multiple European settings, where nearly half of the countries lack specialized adolescent healthcare centers, and policies often fail to address the unique needs of BYM. These gaps underscore the absence of sustainable, equity-driven strategies to close the gender divide in youth clinic access [9].

Sweden, with its publicly funded network of youth clinics, offers a unique context to explore this inequity. Although youth clinics provide SRH, mental health, and general health services, SRH constitutes the majority of visits, which influences both the gendered service identity and the patterns of underrepresentation observed among BYM who constitute only 10–15% of the clinic visitors [10, 11]. The gender imbalance is reinforced by midwifery-dominated staffing and programming, which consolidate a service identity as “girls’ clinics [12].” Young men report feeling out of place and lacking awareness of available services, while providers acknowledge systemic gaps in training, resources, and institutional guidance for engaging them [13]. This pattern is also consistent with extensive international literature showing that reproductive and caregiving responsibilities are culturally constructed as female-gendered labour [14]. Such norms position women and girls as primarily responsible for managing fertility, contraception, and family health, which in turn increases their likelihood of seeking SRH information and services [15].

Internationally, interventions such as adapting waiting spaces and hiring male staff have been linked to improvements in BYM’s service uptake [16]. Few Swedish regional reports highlight efforts by youth clinics to attract BYM, such as hiring male staff to increase male representation in an otherwise predominantly female-staffed environment, adapting service hours, redesigning waiting spaces, and expanding digital outreach to protect privacy [17, 18]. Yet these strategies are implemented with absence of standardized guidance for engaging BYM leading to inconsistent regional efforts [10]. This is concerning given that Sweden’s National Strategy for Sexual and Reproductive Health and Rights (SRHR) explicitly states that “women and men, girls and boys, must have the same conditions for good health and be offered care on equal terms [19].” Without clear policy directives or accountability mechanisms, this commitment risks remaining aspirational.

Although several international studies have examined barriers to boys’ and young men’s engagement in sexual and reproductive health services, systematic mapping of implemented strategies remains rare. Our recent systematic review of interventions aiming at increasing BYM access to SRH services noted that most evidence comes from isolated programmes rather than coordinated national systems [20]. To our knowledge, no country has conducted a national-level mapping of SRH services’ strategies comparable to the present study, positioning this work as a significant contribution to the global evidence base [20].

The lack of systematic mapping of strategies to reach BYM represents an evidence and policy gap with direct equity implications. Failing to address this gap perpetuates gendered disparities in SRH outcomes, undermines the principle of universal access, and limits the potential of youth clinics to serve all young people effectively, specifically men [21]. It also reinforces a narrative in which reproductive responsibility is placed predominantly on girls and young women, rather than promoting a more equitable distribution. By systematically documenting and analyzing strategies to attract BYM, this study seeks to generate actionable insights for policy, service design, and practice. The goal is to advance gender equity in youth health services in Sweden while contributing to the global discourse on inclusive, adolescent-centered care. The aim of this study was to systematically map and analyze the strategies used by Swedish youth clinics to improve access and participation among BYM.

## Methods

### Study design

This study employed an exploratory mixed methods design to identify strategies used by Swedish youth clinics to improve access for BYM. The approach facilitated investigation of a previously underexplored topic. An online survey was distributed to all Swedish youth clinics (September – November 2024), followed by unstructured telephone interviews with consenting participants. Data were analysed using descriptive statistics and qualitative content analysis. This work is part of a larger project on BYM’s access to youth clinics [22].

### Study context

Youth clinics (*ungdomsmottagningar*) in Sweden (~ 240 clinics across 21 regions) provide low-threshold, predominantly free of charge healthcare for adolescents, of all genders, aged 12–25, although the upper age limit varies between regions [10]. Primary services include SRH (e.g., STI testing and treatment), psychosocial support, and health promotion [10]. While 80–90% of attendees are female, clinics aim to increase male utilization of the services [10]. Staff typically include midwives, physicians, psychologists, and social workers [23]. Youth clinic physicians provide care to all genders; no clinics have doctors dedicated exclusively to BYM. Youth clinics are mainly funded by the regions and/or the municipalities and organized under the Swedish Society for Youth Centers (FSUM), an organization that provides guidelines and recommendations for the clinics [24].

### Sampling and recruitment

**Fig. 1:**
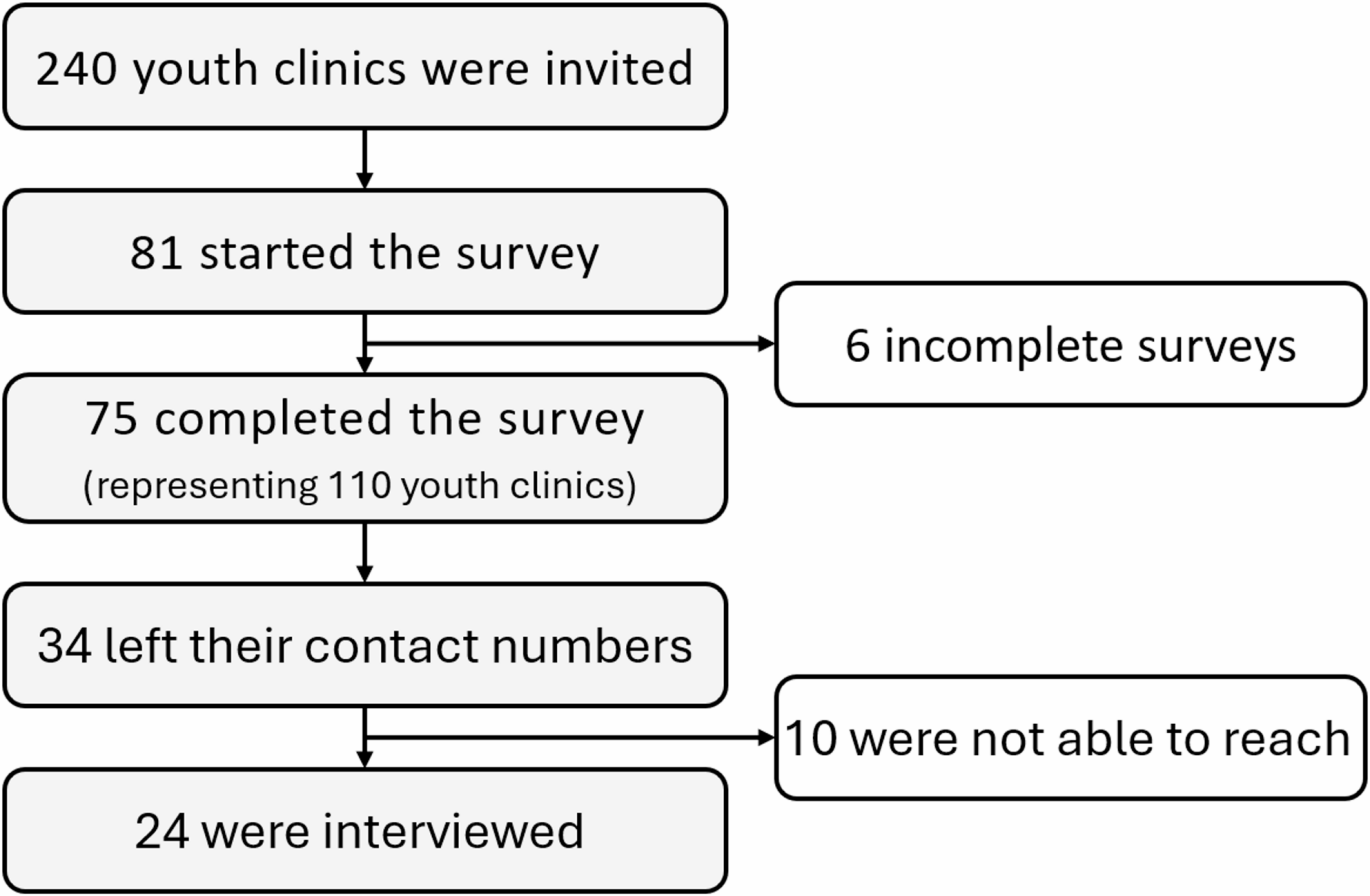
Participant sampling procedure to map and analyse the strategies of youth clinics in Sweden to improve access and participation of boys and young men 2024

All 240 Swedish youth clinics were invited via postal mail (two rounds: 13th and 30th September 2024). Invitations included study information, consent details, and a survey link/QR code. The intended respondents were clinic managers or senior practitioners with operational oversight. All respondents had direct communication and interaction with BYM. Recruitment was supplemented via FSUM’s website and meetings, the project advisory group (which consists of practitioners, managers and operations developers working at several youth clinics in Sweden), and UMSAM (the national network for operation developers and managers within Youth Clinics in Sweden). We asked for one survey to be filled per youth clinic. Of the 240 clinics invited, 81 started the survey and 75 completed the surveys. Since some managers oversaw multiple clinics, the 75 responses represented 110 clinics (45% clinic-level response rate) (see Fig. 1).

### Data collection

Data was collected through an online survey that was open for responses between September and November 2024. The survey was developed by the research team and reviewed by the project advisory group. It included 37 items capturing the clinic’s characteristics and staffing, visitor’s statistics of SRH services and the strategies taken to attract more BYM to youth clinics (for full survey questions, see additional file 1). Follow-up telephone interviews (15–40 min) were conducted with the same managers or senior practitioners who completed the survey and consented to be contacted for an interview. Interviews focused on clarifying the respondent’s own survey responses and exploring the contextual factors shaping strategy implementation; participants were not presented with aggregated survey results. Example questions included: “You mentioned in the survey that you use (strategy), can please elaborate on how it is used in your clinic?” or “Could you clarify what you meant by…”. Unstructured format was chosen to allow flexibility and respondent-driven elaboration. Considering the purpose of the interviews, no audio recordings were made. Instead, detailed notes were taken during and immediately following each call.

### Data analysis

Closed-ended survey items were analyzed descriptively (frequencies/percentages). Qualitative data from open-ended survey responses and interview notes were analyzed using inductive qualitative content analysis following Graneheim and Lundman’s (2004) approach [25]. The process began with immersion in the original Swedish texts to gain holistic understanding. Using NVivo 14 and Excel, all open-ended survey responses were condensed into meaning units and assigned initial codes directly from the text. After coding, all labels were translated into English and similar codes were grouped together. For example, codes such as “anatomical charts” and “condom rooms” were clustered into the subcategory “material/equipment adaptation.” Related subcategories were then organized into four main categories representing strategies used by clinics. The analysis was iterative and refined through discussions within the research team. Preliminary findings were presented to the project advisory group on December 11, 2024, and their feedback informed the final categorization framework.

### Ethical consideration

Ethical approval was obtained from the Swedish Ethical Review Authority (Dnr: 2024-01546-01). Participants provided digital informed consent after reviewing study details, including anonymity, confidentiality, and GDPR-compliant data management. Participation was voluntary, with no incentives provided. The advisory group reviewed survey design and preliminary findings.

## Results

### Quantitative findings

The proportion of BYM visiting SRH services in the clinics was generally low, with a median of 7% and a range between 0% and 30%. The number of BYM visits to SRH services in all participating clinics accounted for 32,202 visits out of total 335,845 visits (9.5%). The participants expressed very limited satisfaction with this level, as 87% reported low satisfaction, 13% moderate satisfaction, and 0% high satisfaction. Perceptions of the clinic’s male-friendliness were somewhat more positive, with 14% rating it as high, 68% as moderate, and 28% as low. A strong perceived need for strategies to attract BYM emerged, with 41% rating this need as high and 53% as moderate, and a believe that the staff would recognise this need was also widespread (42% high, 44% moderate, 14% low). More than half of the clinics (58%) reported that they were currently using strategies to attract BYM, while 22% had done so previously and 21% had never used such strategies. Among those currently applying strategies, 45% reported increased BYM visits, although 55% did not; in contrast, only 10% of clinics not using strategies reported an increase. Evaluation of these strategies remained limited, with just 34% of clinics undertaking evaluations (mainly by looking at the clinic’s statistics), while 66% had not. Levels of institutionalization of these strategies in the clinics’ practices varied, with 33% reporting high institutionalization, 40% moderate, and 26% low. Finally, just over half of the clinics (51%) reported having documents to guide their work with BYM, while 49% did not. (Table 1).

**Table 1:** Quantitative overview of youth clinics’ strategies to improve access and participation of boys and young men in Sweden 2024 (*N* = 75 Participants). BYM: Boys and young men

Metric	Response Distribution
Proportion of BYM visitors to the clinic	Median: 7% (Range: 0–30%)
Satisfaction with BYM proportion in the clinic	High: 0%, Moderate: 13%, Low: 87%
Perceived male-friendliness of the clinic	High: 14%, Moderate: 68%, Low: 28%
Perceived need for strategies to attract BYM	High: 41%, Moderate: 53%, Low: 6%
Staff recognize the need for strategies to attract BYM	High: 42%, Moderate: 44%, Low: 14%
The clinic is using strategies to attract BYM	Currently: 58%, Previously: 22%, Never: 21%
The clinic reported increased BYM visits after implementing strategies to attract them.	For those currently using strategies: Yes: 45%, No: 55% For those not currently using strategies: Yes: 10%, No: 90%
The clinic evaluated the strategies’ effectiveness	Yes: 34%, No: 66%
The clinic institutionalized the strategies	High: 33%, Moderate: 40%, Low: 26%
The clinic has document/s to guide their work with BYM	Yes: 51%, No: 49%

### Qualitative findings

**Fig. 2:**
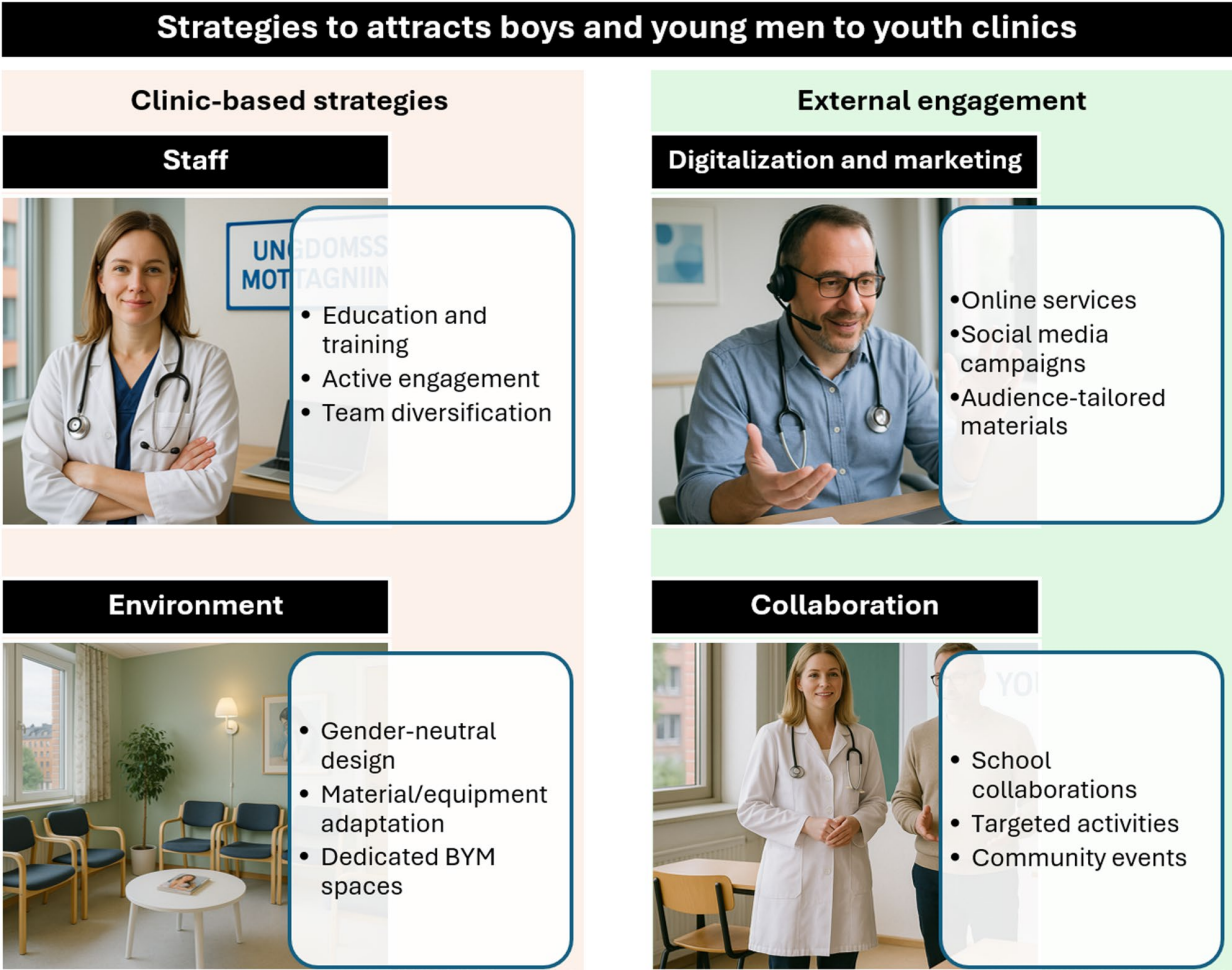
Summary of youth clinics’ strategies to improve access and participation of boys and young men in Sweden 2024 (the pictures in this figure are AI generated). BYM: Boys and young men

Four interconnected categories emerged as the main strategies used to attract BYM to youth clinics in Sweden (Fig. 2). The first two categories are clinic-based strategies: (1) “Staff: enhancing capacity for male-inclusive care” includes efforts to increase staff knowledge and competence related to men’s health, improve interactions with BYM, and hire a gender-diverse team. (2) “Environment: creating inclusive physical and symbolic spaces” highlights strategies to establish an inclusive and neutral atmosphere in waiting and examination rooms, as well as offering different types of receptions. The third and fourth categories focus on external engagement: (3) “Digitalization and marketing: increasing visibility and accessibility” refers to the use of digital tools and marketing strategies to raise awareness and promote clinic use. (4) “Collaboration: reaching BYM where they are” involves efforts to engage more BYM through collaborations with schools and other settings. Each category is presented below with sub-strategies, implementation nuances, and participant voices.

#### Staff: enhancing capacity for male-inclusive care

The staff play a crucial role in making BYM feel safe and welcome. Strengthening staff knowledge about men’s health through targeted training initiatives was highlighted as a key strategy. Many participants described improving staff competence in addressing male health concerns through andrology courses and other training focused on gender roles and treatment. One such example was the “Dockan Dick [Dick the Doll]” workshop, which uses anatomical dolls with interchangeable genitalia to train staff in examining BYM. This type of training was believed to boost staff confidence in clinical encounters and contribute to an increased number of male visitors at youth clinics:

*“We conducted a survey on andrology knowledge among staff*,* and it turned out that many had the knowledge but were very uncertain about the examination process. We put together a training initiative “Dockan Dick”*,* and after the training*,* 80% felt more confident with examination process.” (N85)*.

Active engagement with BYM were also emphasized as an important strategy. Participants noted the importance of making BYM feel seen and heard. Tactics included having a staffed reception area to allow for immediate interaction with drop-in visitors and proactively engaging with BYM in the waiting room by offering conversation or information. Such approaches were seen as effective in creating a positive first impression and making BYM feel more at ease. Staff also described the importance of interacting with BYM who came in to collect free condoms to build trust and foster inclusion:

*“We always have a staffed reception because more boys come spontaneously.” (ID 4).*

“*When boys and young men come to collect condoms*,* we try to prioritize bringing them into the room*,* showing that they are important and have a place at the* clinic*” (ID 59).*

A diverse team comprising various professional roles and gender identities, such as including nurses alongside midwives, was highlighted as an important strategy for providing inclusive care to BYM. While some respondents stated that the gender of the staff did not significantly impact BYM’s experiences, others emphasized that having male staff could foster a greater sense of comfort and belonging. It also allowed BYM the option of speaking with someone of the same gender:

*“We observe that male visitors book appointments with male staff at a much higher rate compared to their female colleagues.” (N65)*.

However, several participants acknowledged the challenges in recruiting male staff for youth clinics, despite a strong interest in increasing gender diversity:

*“I have previously focused a lot on hiring males for the organization*,* but right now we haven’t managed to do that” (N30).*

The perception of midwifery as a female profession that primarily serves women was also identified as a barrier to male engagement. Some participants noted that this perception could make BYM hesitant to seek care. To address this, several clinics emphasized the importance of clarifying that midwives are also trained nurses who play a vital role in men’s health. In some cases, midwives opted to use name tags that identified their profession as “nurse” to help reduce this misconception:

*“Boys and young men do not go to midwives*,* as they believe midwives only cater to women. We have seen an increase in visits now that we have nurses”* (N27).

#### Environment: creating inclusive physical and symbolic spaces

Several youth clinics have made deliberate efforts to create environments that are perceived as neutral and welcoming for all. One common strategy was to use furnishings and artwork that are not gender-coded, aiming to foster an inclusive atmosphere. For example, many clinics redesigned their waiting rooms to be more inviting for BYM, incorporating images and artwork that reflect gender diversity. Displaying anatomical charts of both male and female sex organs was mentioned as a specific tactic:

*“We have a relatively inclusive environment in the waiting room with art that considers gender*,* norms*,* etc.” (N79).*

*“We have consciously tried to tone down signals that can be perceived as gender-coded and consciously highlight what we perceive as gender-neutral. For example*,* anatomical charts of the vulva were previously present in all midwife rooms; now*,* we have added anatomical charts of the penis next to them*,* and anatomical models have also been purchased for all rooms” (N7).*

Some participants also mentioned creating separate dedicated spaces specifically for boys, such as dedicated condom rooms or male-focused consultation rooms:

*“We have a nurse who meets most of the boys. Her room is designed for boys’ needs. But more can be done with*,* for example*,* the other rooms that are more geared towards women’s needs” (N52).*

Adapting both informational materials and medical equipment was another approach used to create a more inclusive environment. Some clinics introduced male anatomical models to support health discussions with BYM and replaced gynaecological chairs with standard examination beds in some rooms to increase comfort and sense of belonging:

*“We try to ensure that we do not signal anything in any way. For example*,* we do not have gynecological chairs in our examination rooms” (N27).*

Despite these efforts, some respondents noted that the physical location of youth clinics could undermine inclusivity. Clinics that were co-located with midwifery or maternity wards were often perceived as less welcoming for BYM:

*“The room is very fertility-focused as it is a room in the maternity ward. Most things in the room scream pregnancy or contraception” (N45).*

Another commonly used strategy was to offer different types of reception services that could lower the threshold for BYM to access care. One frequently mentioned initiative was the introduction of dedicated hours for BYM, referred to as *killmottagning* (“boys’ clinic”) or *POP-mottagning* (“clinics for people with penises and scrotums”). These time slots were usually offered as drop-in sessions but could also be for booked appointments:

*“A men’s clinic has been established with specific times for men to seek help*,* primarily to encourage them to seek help since there are no specific times dedicated to others with different gender identities.” (N65)*.

However, some respondents expressed concerns about gender-specific hours, noting that some BYM found them exclusionary. These participants argued for more integrated services that offer flexibility to all genders:

“*We tested it many years ago. We tried for several years. But when we evaluated it*,* the boys felt it was discriminatory and preferred to come on any day and time that suited them best”* (N61).

#### Digitalization and marketing: increasing visibility and accessibility

Many youth clinics have adopted digital tools and platforms to reach more BYM and make their services more accessible. One such initiative is the launch of online counselling specifically targeted at BYM, referred to as “digital boys’ clinics”, as an alternative to in-person visits. These efforts aim to create more appealing pathways for BYM to engage with the clinics online. Online chat functions were also seen as an effective means of engaging BYM, particularly those who might otherwise not have taken the step to seek help:

*“We will soon open a chat function linked to 1177-direct*,* where it is said that the number of boys will increase significantly. Chatting is intended to reach boys” (N30).*

Another strategy involves offering online booking options for physical BYM’s clinics. Making these services visible online was believed to reduce the threshold for seeking help by increasing awareness and accessibility.

Digital platforms were also used to clearly communicate that BYM are welcome at the clinics and that their health concerns are managed in the clinics. Some clinics launched social media platforms with targeted campaigns, developed online materials addressing common male health issues, and used relatable examples to connect with BYM. For instance, Snapchat was used to publicize drop-in hours for BYM:

*“We have a Snapchat account that regularly informs about this service [boys’ clinic]” (N30).*

Other respondents also pointed to social media’s broader role in communicating male health concerns:

*“We regularly post on our social media with a focus on “boys*,*” e.g.*,* issues related to penis/scrotum*,* mental health problems in boys*,* etc.” (N13).*

Beyond social media, some respondents mentioned producing audience-tailored marketing materials, such as posters and films, that included examples BYM could identify with. These materials were used to increase recognition and awareness of youth clinic services specifically tailored to BYM:

*“We provide many examples of reasons for visits that may suit boys when we conduct outreach work to show the possibilities” (N19).*

These materials were disseminated both online and in physical locations such as schools, universities, and public transport hubs:

*“Surprisingly perhaps*,* but we put up posters at universities stating that there is a boys’ clinic on Thursdays*,* and we have actually seen an increase in the number of boys visiting us.” (N31)*.

A few participants mentioned conducting assessments to understand how well BYM recognized and responded to clinic services, and then refining their branding and outreach strategies based on the findings:

*“In 2021*,* we conducted a target group analysis to find out young people’s awareness and preferences regarding our services in the region. It showed*,* among other things*,* that boys had less awareness of our services. Based on the results*,* we have worked on our visual profile and how we conduct outreach work.” (N33)*.

Understanding youth needs more broadly was also considered important. Some clinics actively sought feedback from youth, including BYM, to ensure their services aligned with users’ expectations:

*“Asked what they want from us. Conducted surveys both at and outside the clinic.” (N61).*

#### Collaboration: reaching boys and young men where they are

Outreach and marketing were widely viewed as essential strategies for connecting with young people. Among these, school visits stood out as one of the most institutionalized and systematic approaches. Most youth clinics offered outreach to both lower and upper secondary schools (*högstadiet* and *gymnasiet*):

*“We largely decide ourselves how we organize our outreach work. We meet all the students in class 8 and 10 once per semester.” (N82)*.

During school visits, clinic staff conducted a range of activities, including sexual education classes, workshops, informal conversations in school corridors to build trust, and even drop-in sessions for STI testing on school premises:

*“One full morning each week*,* we hold drop-in sessions at various high schools*,* with some specifically chosen to reach more boys.” (N19)*.

In some cases, schools were also invited to visit the clinic. This strategy aimed to increase awareness of the clinic’s services and provide students with familiarity regarding the physical space, the staff, and how to access care.

While school outreach initiatives were not necessarily aimed at BYM, they were nonetheless seen as valuable opportunities to engage young men and promote the clinic’s services:

*“We have made special posters that have been sent to all schools in our catchment area*,* informing about our boys’ clinic.” (N30)*.

Several clinics also took more targeted steps to reach BYM, such as increasing their presence in upper secondary schools and vocational programs where BYM are overrepresented:

*“In our city*,* there are many high schools*,* and we have decided that boys should be prioritized*,* so we visit schools where there are more boys.” (N41)*.

In addition to schools, youth clinics extended their outreach to environments where young men tend to gather, such as sports clubs, leisure centres (fritidsgårdar), and vocational training programs:

*“We actively seek out arenas where we know young men are more prevalent*,* such as the military*,* vocational training programs*,* etc.” (N23)*.

*“Since we know that more young men/boys visit the youth leisure center*,* we make sure to be there once per semester.” (N82)*.

Beyond these youth-focused arenas, many clinics collaborated with external actors, such as municipalities, social services, churches, and libraries, to strengthen their community presence. Participation in public events like school graduations or local festivals, including Pride celebrations, was also part of a broader outreach strategy to connect with young people in informal settings:

*“We participate in our local festival every year*,* where we are part of the security team*,* acting as trusted adults for the youth. We also have a tent that young people can visit during the festival.” (N35)*.

## Discussion

This study systematically mapped strategies used by Swedish youth clinics to improve BYM’s access to services. To our knowledge, this is the first national-level study to document and analyze such efforts across Sweden and no country has conducted a national-level mapping of SRH services’ strategies comparable to the present study, positioning this work as a significant contribution to the global evidence base [20]. The findings confirm the existence of multiple promising practices—ranging from staff training to digital outreach and school-based engagement—but also reveal critical gaps in sustainability, institutionalization, and policy coordination. By identifying both existing initiatives and structural barriers, this study extends current understanding of gendered inequities in youth health systems in Sweden and contributes to global debates on how to design gender-responsive, adolescent-centered SRH services.

Clinics reported introducing a range of strategies aimed at making services more welcoming for BYM. These included staff training on men’s health, adjustments to clinic spaces, targeted communication, and school outreach. Similar approaches have been documented in smaller-scale Swedish studies: Thomeé et al. (2016) noted that youth clinics in northern Sweden experimented with extending their activities to reach diverse groups but often lacked systematic evaluation [26]. Internationally, interventions such as adapting waiting spaces and hiring male staff have been linked to improvements in BYM’s service uptake [16]. The present study corroborates these earlier observations while showing that such efforts in Sweden remain piecemeal and lack national coordination.

Capacity-building and digital engagement emerged as particularly promising. Training activities—such as andrology workshops and the use of specialized pedagogical models (e.g., *Dockan Dick*)—were reported to increase staff confidence and normalize discussions with BYM about SRH. This aligns with findings from Waenerlund et al. [27], where professionals highlighted staff preparedness as central to youth-friendly care. Moreover, digital platforms (chats, online booking, and social media campaigns) provided low-threshold entry points that helped bypass stigma and privacy concerns, reflecting global evidence that technology-based outreach is highly acceptable to young men [28–31]. Similarly, school-based health promotion proved valuable, consistent with the World health organization [16], who underscore schools as critical platforms for engaging BYM. Thus, this study confirms the relevance of these strategies in Sweden while demonstrating that their implementation is highly variable across clinics.

Despite these innovations, important gaps remain. Few clinics actively involved BYM in the co-design of services, missing opportunities for peer-led education, referral, and support models. Evidence from other contexts shows that peer involvement can increase trust, reduce stigma, and improve continuity of care [16, 32–34]. Similarly, temporal accessibility—such as evening or weekend hours—was rarely prioritized, despite studies indicating that restrictive opening times are a major barrier for young men who balance school, work, or sports [13, 26, 35]. Engagement of school nurses, who often act as trusted intermediaries for adolescents, was also inconsistent. These omissions highlight a disconnect between global best practices and current practice in Sweden [36].

At a systemic level, there was little evidence of broader structural reforms. Swedish youth clinics lack clear national training standards or accountability mechanisms to ensure male-inclusive services. Without coordinated governance, promising innovations risk remaining isolated and unsustainable.

A key limitation across strategies was their temporary or project-based character. Consistent with Thomeé et al. [26], our findings show that clinics often rely on individual champions or short-term projects rather than embedding innovations into routine practice. Without long-term evaluation or stable funding, promising practices risk being lost or failing to scale. In contrast, global recommendations emphasize institutionalization and sustainability as prerequisites for effective youth-friendly SRH services [16]. The lack of evaluation also restricts the evidence base needed for policy advocacy and service redesign.

Although youth clinics are usually perceived as highly youth friendly [27, 37, 38], this study highlights that Swedish youth clinics continue to operate primarily within a gender-neutral framework, formally open to all but insufficiently attentive to BYM’s unique barrier [26, 35]. Literature has consistently cautioned that gender-neutral approaches can reinforce inequities by assuming that equal access equates to equal use [39]. BYM’s perceptions of clinics as “girls’ spaces,” shaped by midwifery-led traditions, mirror findings from earlier studies in Sweden [21, 35] and echo broader European evidence of female-oriented service environments. Moving toward a gender-responsive framework—explicitly addressing the cultural and structural barriers BYM face—is therefore essential [16]. Globally, gender-responsive approaches have been linked to more equitable SRH outcomes [40]. This study’s findings reinforce calls for Sweden to reorient its youth clinics toward such a framework, in line with national commitments to SRHR equity.

By documenting strategies across nearly half of all Swedish youth clinics, this study provides the most comprehensive mapping to date of efforts to engage BYM. Its key contribution lies in demonstrating that while there is strong recognition of the problem at the clinic level, responses remain fragmented and lack systemic support. Unlike earlier regional studies by Thomeé et al. and Waenerlund [26, 27], this national-level perspective highlights the structural nature of the inequity and the urgent need for coordinated policy action. Specifically, national guidelines, standardized staff training, and equity monitoring frameworks could provide consistency and sustainability. Such reforms would align Sweden with global recommendations on adolescent health and gender and prevent BYM from continuing to be underserved within an otherwise universalist health system. Future research could explore how boys and young men perceive youth clinics, including their awareness of available services. Understanding their perspective could provide valuable insight for policy development.

### Strengths and limitations

This study is the first to systematically map strategies aimed at addressing the gender gap in access to youth clinics in Sweden, offering a broad and diverse snapshot of current practices. However, the response rate was relatively low (∼45% of clinics). It is likely that respondents had a particular interest or engagement in improving BYM access, which may skew the findings toward more proactive or innovative practices. This limits the generalizability and transferability of the results to all youth clinics in Sweden. Another limitation is that visit data were only related to SRH services in youth clinics and not mental health, or other services, preventing analysis of whether BYM underrepresentation varies between SRH and other services. Additionally, the heterogeneity and limited granularity of clinic‑level variables, made inferential statistical comparisons (e.g., between clinics with higher vs. lower BYM proportions) not feasible.

The use of both quantitative survey data and qualitative interview insights provided breadth as well as depth in understanding strategy implementation, institutionalization, and perceived effectiveness. Credibility and relevance were further strengthened by review of the preliminary findings by a project advisory group composed of youth clinic professionals.

Some of the responses were short or described strategies indented for all youth, rather than specifically tailored to BYM, however, data were collected from clinic managers and senior practitioners with operational oversight, ensuring that responses reflected grounded, practice-based perspectives. At the same time, most of the reported strategies had not been formally evaluated, making it difficult to assess their actual impact on BYM utilization of clinic services.

## Conclusions

This study demonstrates that BYM remain significantly underrepresented in Swedish youth clinics, with most clinics dissatisfied with current levels of engagement. While many clinics have piloted strategies to address this gap, these are often temporary, inconsistently applied, and rarely evaluated. The absence of national guidelines, training standards, and accountability structures has resulted in fragmented and unequal services across regions.

The findings underscore that gender-neutral approaches are insufficient and risk reinforcing existing inequities. A shift toward gender-responsive care—anchored in policy, training, and service design—is urgently required. Priorities include involving BYM in the design of services, expanding access through temporal flexibility and peer-led models, and embedding male-inclusive practices into routine clinic operations. At the policy level, stronger coordination, national training standards, and systematic equity monitoring are essential.

Overall, this study contributes new evidence on the strategies and limitations of engaging BYM in Swedish youth clinics. Its findings highlight the urgent need for long-term, coordinated, and gender-responsive approaches to ensure that BYM can benefit equally from services intended to promote adolescent health and wellbeing.

## Supplementary Information

Supplementary Material 1.

## Data Availability

The datasets generated and/or analysed during the current study are not publicly available due to institutional data protection policies and confidentiality agreements with participating clinics, but are available from the corresponding author on reasonable request.

## Abbreviations

BYM: Boys and Young Men
FSUM: Swedish Society for Youth Centers
SRH: Sexual and Reproductive Health
SRHR: Sexual and Reproductive Health and Rights
UMSAM: The National Network for Operation Developers and Managers within Youth Clinics in Sweden
