# Supplementary material for: Mapping the strategies to address the gender gap in youth clinic utilization in Sweden; explorative survey and follow up interviews
Source: Arch Public Health. 2026 Apr 24;84:94. doi: 10.1186/s13690-026-01918-y (PMC13112811; doi:10.1186/s13690-026-01918-y)
Supplement: Supplementary file 1 — Supplementary Material 1. [file 13690_2026_1918_MOESM1_ESM.docx]

**Survey: Bridging the gap between the youth clinics and young men**

**The clinic**

1. What is the name of your clinic?

2. What is the upper age limit for visiting your clinic?

3. Who is responsible for financing the clinic?

- Private operator
- Region/county
- Municipality

**Staff overview**

4. How many midwives work at your clinic (counted as full-time equivalent)?

5. How many counselling staff (counsellor, social worker, psychologist, etc.) work at your clinic (counted as full-time equivalent)?

6. How many doctors work at your clinic (counted as full-time equivalent)?

7. How many of the staff identify as men?

8. How many of the staff have andrological training/competence?

**Statistics**

9. How many visits have your clinic had in the last 12 months?

10. How many visits in the last 12 months were by boys and young men?

11. How many unique individual visits have your clinic had in the last 12 months?

12. How many unique individual visits in the last 12 months were boys and young men?

**Strategies and measures**

13. How satisfied are you with the proportion of boys and young men at your clinic at present? Please rate on a scale from 0 to 10 (0 = least satisfied; 10 = most satisfied).

14. What are the most common reasons for visits among boys and young men?

15. To what extent do you consider the environment at your clinic to be male-friendly? Please rate on a scale from 0 to 10 (0 = least friendly; 10 = most friendly)

*Male-friendly services are those that can meet the unique needs and preferences of male users.*

16. Can you explain the rating you gave in the previous question?

17. Do you see any potential advantages or disadvantages to increasing the use of your clinic by boys and young men?

18. Does your clinic offer special services exclusively for boys/young men, such as special appointment times? If so, which services?

29. What outreach work does the clinic have and how many hours the staff can devote to this work?

20. Have you previously used any strategy to increase the use of your clinic by boys and young men?

- Yes
- No

21. Do you currently use any strategies to increase the use of your clinic by boys and young men?

- Yes
- No

22. If yes to either of the above questions, explain in as detail as possible what measures/strategies the clinic took.

23. To what extent are these measures/strategies routinized in your current practice? Please rate on a scale from 0 to 10 (0 = low extent; 10 = high extent).

24. If these measures/strategies have been discontinued/interrupted, please explain why.

25. Have the results been measured/followed up?

- Yes
- No

26. If so, what tools have been used?

27. Can you, for example, write down the three most significant results of these measures?

28. Are/were these measures/strategies effective or not? Please justify your answer.

29. Has the number of male adolescents increased?

- Yes
- No

30. If yes, by approximately how much has it increased?

31. On a scale of 0 to 10, how much do you think that your clinic needs measures/strategies to attract more boys and young men to your clinic? (0 = least need; 10 = greatest need).

32. How do you think the clinic’s staff will perceive the need to take these measures/strategies? Please rate on a scale of 0 to 10 (0 = least need; 10 = greatest need).

**Improvements**

33. Is there any document (national, regional or local) that guides your work with boys' and young men's SRH and their access to the clinic?

- Yes
- No

34. If yes to the above question, please provide a link to this document or upload it to the survey.

35. Are there any suggestions or anything else you would like to share regarding how to improve boys' and young men's use of youth clinics?

36.If we need more information/additional information, would it be okay to follow up with you by phone?

- Yes
- No

37.Please provide your name and contact number/email address, as well as the best times and days of the week to reach you.
